# Isolation and Characterisation of PRSV-P Resistance Genes in *Carica* and *Vasconcellea*

**DOI:** 10.1155/2014/145403

**Published:** 2014-08-11

**Authors:** M. R. Razean Haireen, R. A. Drew

**Affiliations:** ^1^Malaysian Agricultural Research and Development Institute, Persiaran MARDI-UPM, 43400 Serdang, Selangor, Malaysia; ^2^Griffith University, Nathan Campus, 170 Kessels Road, QLD 4111, Australia

## Abstract

Papaya (*Carica papaya* L.) is one of the major tropical fruit crops worldwide, but it is limited throughout its range by papaya ringspot virus type P (PRSV-P). Previous genetic studies identified a functional PRSV-P resistance marker in a mapping population of F_2_ plants of *Vasconcellea pubescens* (resistant to PRSV-P) × *Vasconcellea parviflora* (susceptible to PRSV-P) and showed that the marker exhibited homology to a serine threonine protein kinase (STK) gene. Full length cDNAs of putative PRSV-P resistance genes designated CP_STK from *C. papaya* and VP_STK1 and VP_STK2 from *V. pubescens* were cloned by rapid amplification of cDNA ends (RACE). Due to a frame-shift mutation, the two homologous sequences are transcribed and edited differently such that the gene product in *V. pubescens* is two separate transcripts, whereas in *C. papaya* they are fused into a single message. A peroxisomal targeting signal (PTS2) present in VP_STK2 but absent in the other transcripts may be the functional source of PRSV resistance in *V. pubescens*. The STK gene from *V. pubescens* may have been derived from an alternative splicing to confer resistance. The putative resistance gene, *VP_STK2*, that was identified in this study is a potential new source of PRSV-P resistance for papaya genotypes.

## 1. Introduction

Papaya ringspot virus type P** (**PRSV-P) is a devastating disease of papaya that affects tree vigour, fruit set, and quality [1]. Once infected, the fruit yield and plant's productive life are reduced from three years to one year or less [2]. In Australia PRSV-P is considered a serious threat to the Australia papaya industry even though it has not occurred in the major growing region of North Queensland [3].

Breeding for resistance to PRSV-P in papaya has resulted in tolerant varieties only [4, 5] because no resistance to PRSV has been discovered within the* Carica *genus. In practical plant breeding programs, genes for plant disease resistance are frequently identified in noncommercial, wild plant relatives and introgressed into commercially acceptable cultivars. Introgression of resistance genes from* Vasconcellea quercifolia* to* C. papaya* is an example of intergeneric hybridization that has been used to develop partial resistance to PRSV-P in* C. papaya *[6].

Dillon et al. [7] identified a potential functional sequenced characterised amplified region (SCAR) marker (Opa11_5r) for PRSV-P resistance which collocated with* prsv-1*, from a mapping population of wild relative of papaya, F_2_ *V. pubescens* ×* V. parviflora*. Dillon [8] characterized the Opa11_5r marker in six* Vasconcellea* species (*V. pubescens*,* V. stipulata*,* V. goudotiana*,* V. cauliflora*,* V. parviflora,* and* V. quercifolia*) as well as in* C. papaya* genotype 2.001. The marker had homology to a serine threonine protein kinase (STK). STK is one of the important proteins responsible for defence signal transduction related to resistance of a range of plant pathogens including viruses and was reported to be involved in disease resistance in other crops [9].

Peroxisome targeted protein kinase is suspected to mediate signal transduction across the membrane.

Proteins destined for peroxisomes are known to be synthesized with a peroxisomal targeting signal (PTS). There is two peroxisomal targeting signals: PTS1 as a 9-amino acid sequence at the N-terminal of the protein and PTS 2 as a tripeptide at the C-terminal.

In the disease resistance study, signal transduction comprises the transmission of extracellular signals to intracellular responses. An extracellular domain could be required to bind several ligands. Ligand binding induces a conformation change in the extracellular domain which is hypothesized to result in dimerization and bringing the intracellular kinase domains into close proximity [10]. That multifactor binding event is followed by transmission of secondary signals through the plasma membrane. The accumulation of intracellular signalling from the receptor causes the induction of specific phosphorylation cascades. Phosphorylation of protein kinases can be involved in either direct interaction with the pathogen or downstream signaling leading to expression of defence-related genes [11].

In studies on plant viruses, viral-encoded proteins are reported to suppress plant kinase activity through specific binding to a kinase domain and enhance the pathogenicity of the virus. Protein-protein interaction of AL2 from tomato golden mosaic virus (TGMV) and L2 from beet curly top virus (BCTV) is amongst those reported to inhibit sucrose nonfermenting 1- (SNF1-) related kinase activity in plants [12]. However, signalling through phosphorylation of cellular and virus protein has been shown to modulate symptoms expression and pathogenicity [13]. Protein signalling through phosphorylation of viral-encoded protein promotes translocation of the ribosomal protein to the nucleus where it may negatively impact virus infection [13].

There has been considerable speculation about the plant component of a gene-for-gene interaction and the products of natural resistance genes [14, 15] in plant resistance. Genomic analysis and characterization of papaya's resistance genes [16] and those of its wild relatives are important to provide additional sources of resistance for* C. papaya* improvement. We postulated that Opa11_5r marker could be a sequence region in a complete STK gene of* C. papaya* and* V. pubescens* and thus important to determine the polymorphism between* C. papaya* and* V. pubescens*. This study characterizes the transcripts from the orthologous STK genes in PRSV-susceptible* C. papaya* and PRSV-resistant* V. pubescens* to determine whether structural differences exist, and if so, how they may relate to the difference in virus disease resistance.

## 2. Materials and Methods

### 2.1. Plant Materials

*C. papaya* (genotype 2.001) and* V. pubescens *plants were maintained in tissue culture at Griffith University, Nathan campus. Plants which grew from shoot tips of plants maintained in tissue-culture were micropropagated* in vitro *using methods described by [17]. Plants were incubated under light/dark conditions of 16-hour photo period illuminated by white fluorescent lamps and eight hours of darkness at 25°C ± 1°C.

### 2.2. First Strand RACE cDNA Synthesis and Amplification of STK Full Length cDNA from* C. papaya *and* V. pubescens*

Total RNA was extracted from the leaves using a Nucleospin RNA plant kit (Macherey-Nagel) according to the manufacturer's protocol. The two genes amplified in this study are labelled here as “106” and “105” according to application of automated gene prediction models to the* C. papaya* whole genome sequence that is available in ftp://asgpb.mhpcc.hawaii.edu/papaya/. For synthesis of 5′-RACE cDNA, a 100 ng of total RNA was reverse-transcribed with 5′-RACE CDS primer A and 1.2 *μ*M SMARTER II A oligonucleotide by a 10 U SMARTScrib Reverse Transcriptase (Clontech). For synthesis of 3′-RACE, 100 ng of total RNA was reverse transcribed with 3′-RACE CDS primer by a 10 U SMARTScrib Reverse Transcriptase (Clontech).

The 5′-RACE and 3′-RACE PCR reaction were conducted to amplify the* STK106* gene of* C. papaya* and* V. pubescens* in accordance with the protocol provided by the manufacturer of an Advantage 2 PCR Kit (Clontech) with Universal primer A Mix and 0.2 *μ*M gene-specific primer; gsp1 and gsp2 in 5′ and 3′-RACE PCR respectively (Table 1). The PCR procedure was conducted under the following conditions: 4 min at 94°C, 5 cycles (30 s at 94°C, 3 min at 72°C), 25 cycles (30 s at 94°C, 30 s at 60°C, 3 min at 72°C) and 5 min at 72°C.

Nested-PCR reaction was performed as a secondary reaction in cases where the primary PCR failed to give the distinct band of interest or produced a smear. In this study, nested-PCR was further performed for* STK106* gene amplification from* C. papaya* and* V. pubescens* using a diluted 3′-RACE-PCR and 5′-RACE-PCR product with gene-specific primer; ngsp2_106 and ngsp1_106 respectively (Table 1).

Similar* to STK106* gene, the first strand cDNA synthesis and amplification of* STK105* gene of* C. papaya* and* V. pubescens* were conducted in accordance with the protocol provided by the manufacturer of an Advantage 2 PCR Kit (Clontech). The RACE-PCR procedure was the same as that described for the* STK106* amplification except that the specific primer; gsp1_105 and gsp2_105 was used in 5′ and 3′-RACE PCR respectively (Table 1). The amplified product of* C. papaya* and* V. pubescens* was purified and cloned into pCR8/GW/TOPO vector (Invitrogen) according to the manufacturer's protocol. Plasmid DNA was extracted using PureLink Quick Plasmid Miniprep kit (Invitrogen) according to the manufacturer's protocol and sequenced by the dideoxy chain termination method of [18] using the BigDye version 3.1 sequencing system (Applied Biosystem). The sequencing products of 5′ and 3′ RACE were then aligned and assembled to deduce the full length cDNA.

### 2.3. Characterization of the STK Gene in* C. papaya* and* V. pubescens*

Sequence alignments, ORF translation and predicted protein were carried out using Expasy translate tools (http://web.expasy.org/translate/). Sequences homology were identified from the sequence database using Basic Local Alignment Tool [19] supported on the website (http://www.ncbi.nlm.nih.gov/) of the National Centre for Biotechnology Information (NCBI). Searches for regions locally similar to nucleotide and protein were initiated using BLASTn and BLASTx tools respectively.

Protein subcellular localization prediction was carried out using Wolf psort (http://wolfpsort.org/). Protein subcellular localization prediction was carried out using Wolf psort (http://wolfpsort.org/). Transmembrane prediction using Hidden Markov Models (TMHMM) in a protein was derived using http://www.cbs.dtu.dk/services/TMHMM-2.0. A prediction of membrane spanning regions and their orientation was carried out using TMPred (http://www.ch.embnet.org/). Identification of the protein domains, families & functional sites and associated patterns & profiles were carried out using Expasy-prosite (http://prosite.expasy.org/).

## 3. Results

### 3.1. Isolation and Analysis of STK Full Length cDNA of* C. papaya*

The nested-PCR performed using a diluted* C. papaya* 3′-RACE-PCR product with nested universal primer (Clontech) and gene-specific primer: ngsp2_106 [8] produced a 1014 bp fragment. This fragment was sequenced and used to design a gene-specific primer, ngsp1_106. Nested-PCR was performed using a diluted* C. papaya* 5′-RACE-PCR product with nested universal primer (Clontech) and gene-specific primer; ngsp1_106 produced an 843 bp fragment. The amplified forward and backward fragments of nested-PCR were cloned and assembled to determine the full length cDNA which was named* c28.106*.

Alignment of the open reading frame (ORF) to the online* C. papaya *ORF (ftp://asgpb.mhpcc.hawaii.edu/papaya/) showed that* c28.106* was homologous to gene* 106 *in supercontig 28 (*28.106*), sized 1016 bp, and encodes for a serine threonine protein kinase (STK) gene. Nevertheless the deduced amino acid sequence in* c28.106* or* 28.106* revealed no stop codon in the C-terminal region.

When aligned,* 28.106* showed a high homology in the upstream coding region of* c28.106*. The downstream region of* c28.106* was homologous to an adjacent gene,* 105* in supercontig 28,* 28.105* (online* C. papaya *ORF; ftp://asgpb.mhpcc.hawaii.edu/papaya/) which also encodes for a STK gene. Therefore, the sequence* 28.105* was used to design specific primers, gsp2_105 in 3′-RACE-PCR and gsp1_105 in 5′-RACE-PCR. The amplification with universal primer A mix (Clontech) and gene-specific primer: gsp2_105 in 3′-RACE-PCR revealed a 998 bp fragment. Amplification with universal primer A mix (Clontech) and gene-specific primer: gsp1_105 in 5′-RACE-PCR revealed a 480 bp fragment. The amplified forward and backward fragments of RACE-PCR were cloned and assembled to determine the nucleotide sequence of the full length cDNA which was named* c28.105*. The full length sequence (which is verified by the presence of start and stop codons) of* c28.105* showed a 100% similarity in the 3′ flanking region to* c28.106*. This result suggested that* c28.106* and* c28.105* existed as one gene that encodes for a STK in* C. papaya* rather than being two different genes.

The full length cDNA sequence which is characterized with polyadenylation signal, AATATA in the 3′ flanking region at the position 219 nucleotides from the TGA termination signal, was determined and designated as* CP_STK*. It has been registered in National Centre for Biotechnology Information (NCBI) with Accession number KC310466.* CP_STK* sized 2520 bp, containing a 1617 bp open reading frame (ORF) encodes for 539 amino acids, a 5′-untranslated region of 371 bp, and a 3′-untranslated region of 532 bp. The physicochemical properties of CP_STK whole protein are shown in Table 2. BLASTp analysis showed that CP_STK had homology to* STK* in other species:* Glycine max* (Accession number: XP003547484),* Ricinus communis *(Accession number: XP002514097), and* Vitis vinifera *(Accession number: XP002279199).

The amino acid sequence analysis showed that CP_STK of papaya had a protein kinase domain in the upstream region at position 114 to 421 and an AGC_kinase C terminal domain in the downstream region at position 422 to 494. Analysis for the transmembrane showed that CP_STK did not have transmembrane helices. The protein subcellular localization prediction analysis showed that CP_STK had an endoplasmic reticulum (ER) signal, DKRA which described that the protein synthesized by ribosomes remains suspended in cytosol. Rather than that, CP_STK did not have a second peroxisomal targeting signal in its sequence to explain that it is not destined for peroxisomes.

### 3.2. Isolation and Analysis of STK Full Length cDNA of* V. pubescens*

The mRNA transcript region sequences in* V. pubescens *cDNA clones were confirmed with the primers that were used in* C. papaya. *The nested-PCR performed using a diluted* V. pubescens* 3′-RACE-PCR with nested universal primer (Clontech) and gene-specific primer: ngsp2_106 [8] produced a 1770 bp fragment. The nested-PCR performed by diluted 5′-RACE-PCR product with nested universal primer (Clontech) and gene-specific primer: ngsp1_106 produced a 1059 bp fragment. The amplified forward and backward fragments of nested-PCR were cloned and assembled to obtain the full length cDNA that was 2524 bp long and was designated as* VP_STK1*. The* V. pubescens *cDNA contained a 921 bp ORF with a 5′-untranslated region of 587 bp and a 3′-untranslated region of 1016 bp. A polyadenylation signal, AATATA, was present, 920 nucleotides from the TGA termination signal.

Subsequently, the same gene-specific primers as used for* C. papaya* were used in the amplification of gene* 28.105* in* V. pubescens*. The amplification with universal primer A mix (Clontech) and gene-specific primer: gsp2_105 in 3′-RACE-PCR revealed a 1601 bp fragment. Amplification with universal primer A mix (Clontech) and gene-specific primer: gsp1_105 in 5′-RACE-PCR revealed a 1059 bp fragment. The amplified forward and backward fragments of RACE-PCR were cloned and assembled to obtain the full length cDNA which was designated as* VP_STK2*. The gene was 1664 bp in length, contained a 582 bp open reading frame that encoded for 194 amino acids, and had a 5′-untranslated region of 389 bp and a 3′-untranslated region of 693 bp. Polyadenylation signal, AATATA, was present 218 nucleotides from the TGA termination signal. The physicochemical properties of VP_STK1 and VP_STK2 whole protein are shown in Table 2.

The full length cDNA sequence for* VP_STK1* and* VP_STK2* was not identical when they were aligned. In contrast to the homologous* C. papaya *gene, a stop codon (TAA) was identified at position 1506 to 1508 bp in* VP_STK1*. This resulted from one nucleotide deletion at position 1495 bp. The gene was predicted to be spliced and encode for two* STK* genes. This result confirmed that* VP_STK1* and* VP_STK2* were separate as adjacent transcripts in* V. pubescens* and different from the orthologous gene in* C. papaya*. Both* VP_STK1* and* VP_STK2* have been registered in NCBI with accession numbers KJ489312 and KJ 489313, respectively.

VP_STK1 and VP_STK2 had homology to* STK* in other species:* Ricinus communis *(Accession number: XP002514097),* Glycine max* (Accession number: XP003547484),* Medicago truncatula* (Accession number: XP003595251), and* Vitis vinifera* (Accession number: XP002279199).

The amino acid sequence analysis revealed a protein kinase domain in VP_STK1 at the amino acid position 109 to 306 and an AGC_kinase C terminal domain in VP_STK2 at the amino acid position 74 to 144. TMHMM analysis for the transmembrane showed that VP_STK1 and VP_STK2 did not have transmembrane helices. Nevertheless VP_STK2 did have signal peptide, putative cleavage site after amino acid position 13. The protein subcellular localization prediction analysis showed that VP_STK1 had an endoplasmic reticulum (ER) signal, DKRA, and did not have a second peroxisomal targeting signal similar to CP_STK. By contrast, VP_STK2 did not have an endoplasmic reticulum (ER) signal but did have the second peroxisomal targeting signal (KIVHWRHHL) at amino acid position 22.

**Figure 1:**
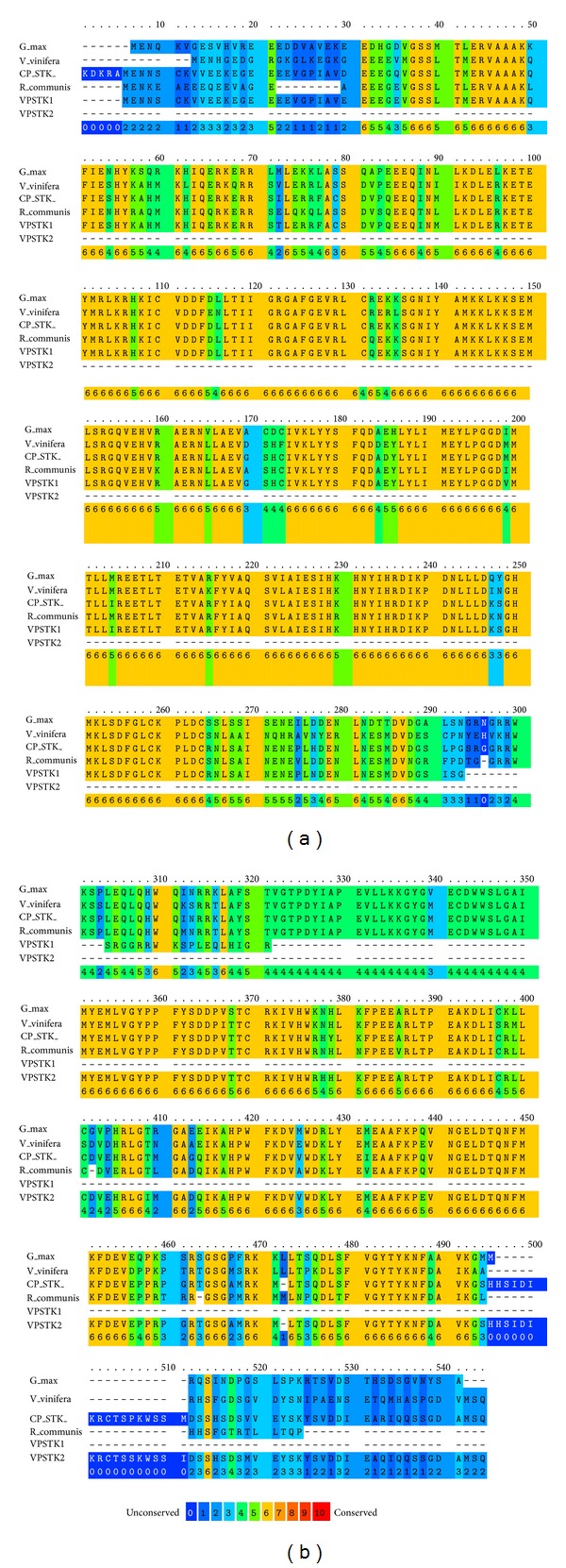
Alignment of deduced amino acid sequences of CP_STK, VP_STK1, and VP_STK2 with the sequences of STK* Ricinus communis* (XP002514097),* Glycine max *(XP003547484), and* Vitis vinifera* (XP002279199). Unconserved to conserved region is coloured in scale from 0 to 10 and shown on the last line in each paragraph.

Alignment of deduced amino acid sequences of CP_STK, VP_STK1, and VP_STK2 to the sequences of STK* Ricinus communis* (XP002514097),* Glycine max* (XP003547484), and* Vitis vinifera* (XP002279199) is shown in Figure 1. Unconserved to conserved regions are coloured in scale of 0 to 10. The amino acid sequences are highly conserved in the middle region of the sequence. VP_STK1 is conserved at the upstream region while VP_STK2 is conserved at the downstream region when compared to the other clones and species.

## 4. Discussion

The upstream coding region of cDNA transcript,* CP_STK,* from papaya genotype 2.001 was 100% similar to papaya genomic sequence 28.106. Nevertheless* CP_STK* was longer than* 28.106* in that the downstream region of* CP_STK* showed similarity to another kinase gene in the* C. papaya* genome sequence,* 28.105*. A structural difference is evident between the cDNA and genomic sequences. A longer STK gene in* C. papaya* genotype 2.001 was expected as no stop codon was found in the nucleic acid sequence of* 28.106* available in ftp://asgpb.mhpcc.hawaii.edu/papaya/. Based on this result, a new* STK* gene in* C. papaya* has been registered in NCBI under accession number KC310466.

TMHMM analysis for the transmembrane protein showed that CP_STK, VPSTK_1 and VP_STK2 did not have transmembrane helices. Nevertheless, the amino acid sequence analysis showed that CP_STK and VP_STK2 had an AGC_kinase C terminal domain in the downstream region. The AGC (cAMP-dependent, cGMP-dependent, and protein kinase C) is known as AGC kinase C terminal. The AGC in the protein kinase family contains a collection of protein kinases that display a high degree of sequence similarity within their respective kinase domains with phosphorylation sites.

Although CP_STK and VP_STK2 could be phosphorylated, but the presence of signal peptide in VP_STK2 targets it for secretion and a C-terminal extension. This is supported by a report on* Arabidopsis thaliana* that showed the* RPP5* gene is cytoplasmically localized, as it had no signal peptide or membrane spanning region in its gene sequence, “as reviewed by [20].”

Sequencing of mRNA from the STK gene of the resistant parent,* V. pubescens*, revealed two discrete transcripts of the same gene. These two transcripts represent the first and last sections in the* STK* gene of* C. papaya/R. communis* (Accession number: XM_002514051), which are separated in* C. papaya* by a 20 kb intron. Sequence differences between the orthologous genes from* C. papaya* and* V. pubescens* were expected to encode for a different protein function and expression. These differences may be the reason that* V. pubescens *is resistant to PRSV-P when compared to* C. papaya* that is susceptible. The isolated* 106* gene in* V. pubescens*, named* VP_STK1*, was orthologous to the* STK* gene and had 95.66% similarity to* 28.106* in* C. papaya*. The conserved region was found mostly at the 5′-end. An “activation loop” is located in exon 5 and the start of exon 6 prior to the premature truncation of the* VP_STK1* gene. Kinase activity could be increased when a residue on the activation loop close to the catalytic center is phosphorylated. The isolated* 105* gene, named* VP_STK2*, was orthologous to the* STK* gene in* C. papaya*. The predicted start of the* VP_STK2* gene is at a start codon (Met) that truncates six codons from the seventh exon of the* C. papaya* gene. This was supported by an alignment result of nucleotide sequences of* c28.106* of* C. papaya* with* VP_STK1* of* V. pubescens*. A deletion of one base pair of nucleotides in* VP_STK1* of* V. pubescens* was observed when compared to the sequence in* C. papaya*.

Based on supercontig 28 of the* C. papaya* genome sequence, there are very large introns in the gene, which would probably encourage alternative splicing. These genes are adjacent in* V. pubescens* and the orthologous gene in* C. papaya*/*R. communis* spans both of these* V. pubescens *genes. The* VP_STK1* gene is predicted to be separated from* VP_STK2* because the sequence has a STOP codon in what is otherwise the middle of one of the exons of the* C. papaya*/*R. communis* gene. The predicted end of the* VP_STK1* gene is a stop codon that is not found in the* C. papaya* or* R. communis* genes, truncating 39 bp (13 codons) from the sixth exon.

Inside the nucleus, splicing takes place in a process called posttranscription modification before the mRNA can be decoded by ribosomes to produce a protein whilst in alternative splicing, two or more different mature mRNAs are decoded by ribosome to produce multiple proteins. Alternative splicing predicted in this study is strongly supported by the result of [8] who found another marker, Opk4_1r, that was close to the* prsv-1* resistance gene in* V. pubescens* but not in* C. papaya*. Opk4_1r has homology to a gene that codes for a small nuclear ribonuclear class of protein (snRNP) which has a motif known as a RNA binding domain (RBD) or ribonucleoprotein (RNP). This class of proteins is involved in the posttranscriptional gene expression processes including mRNA and rRNA. Spliceosome, a large ribonucleoprotein (RNP) complex that contains small nuclear RNP particles, snRNP, and other numerous protein factors including RNA helicases and protein kinases are involved in the splicing process in plants [21, 22]. Products of alternative splicing are significant in cellular functions including signal transduction, immunity, disease resistance, transport, regulation, and development [23].

In this study, VP_STK2 that presumed as a protein variants produced by an alternative splicing in* V. pubescens*, could be imported and ultimately resides within peroxisome, as a second peroxisomal targeting signal (PTS2) signal was found in the N terminus of VP_STK2 but not in CP STK or VP_STK1. PTS is a region of the peroxisomal protein that recognises and binds to the receptor. This is supported by a few peroxisomal membrane proteins such as plant APX and a viral protein that are known to be delivered to peroxisomes via distinct ER subdomains [24, 25]. Reference [26] in 2007 reported their proteome data that support the function of plant peroxisomes against pathogens. Furthermore, the existence of protein kinases and phosphatases in plant peroxisomes has been reported by [27]. Different from CP_STK and VP_STK1, their mRNA is presumed to move through the nuclear pore into the cytoplasm and code into a protein. The proteins synthesized by ribosomes then become attached to the membranes of the endoplasmic reticulum (ER). This is supported by the presence of endoplasmic reticulum (ER) membrane retention signal and the absence of PTS2 signal in both of the proteins.

## 5. Conclusion

The findings in this study confirmed and fully supported that hypothesis of the variations of the gene (from resistant to susceptible) was due to structural differences of the serine/threonine protein kinase sequence. An alternative splicing that occurs in* V. pubescens* mRNA and the presence of a peroxisomal targeting signal (PTS2) in VP_STK2 are hypothesised to be an important factor in contributing to the PRSV-P resistance in* V. pubescens*. Nevertheless a further biochemical analysis and gene transformation studies in the future will enable clarification and confirmation of the involvement of VP_STK2 in transferring the PRSV-P resistance from* V. pubescens* to* C. papaya*.

## Figures and Tables

**Table 1:** Primer sequence used in RACE-PCR and nested-PCR.

Primer	Forward-primer	Reverse-primer	Targeted genes to be amplified
gsp2	AATCGCCGTAGAGGAGGAGG		*28.106 (STK106) *
gsp1		AATCGCCGTAGGAAAATTC	
ngsp2_106	GCATATCCAAGAACGCAAGG		
ngsp1_106		TCTCCGCCCGAACATGTTCAACC	

gsp2_105	TGAAATTTGATGAGGTGGAACCTCC		*28.105 (STK105) *
gsp1_105		TGATGCGAACCTTTGACAGC	

**Table 2:** Physicochemical properties of whole protein.

Protein designation	Accession number	Amino acid	Molecular weight (Da)	Isoelectric point (pI)	ORF (bp)	5′-UTR (bp)	3′-UTR (bp)
CP_STK	KC310466	539	62002.6	5.93	1617	371	532
VP_STK1	KJ489312	307	35860.9	5.86	921	587	1016
VP_STK2	KJ489313	194	27428.0	5.72	582	389	693
